# Validation and marker-assisted selection of DArT-genomic regions associated with wheat yield-related traits under normal and drought conditions

**DOI:** 10.3389/fgene.2023.1195566

**Published:** 2023-05-23

**Authors:** Mostafa Hashem, Karansher Singh Sandhu, Saleh M. Ismail, Andreas Börner, Ahmed Sallam

**Affiliations:** ^1^ Department of Genetics, Faculty of Agriculture, Assiut University, Assuit, Egypt; ^2^ Bayer Crop Sciences, Chesterfield, MO, United States; ^3^ Soils and Water Department, Faculty of Agriculture, Assiut University, Assiut, Egypt; ^4^ Leibniz Institute of Plant Genetics and Crop Plant Research (IPK), Gatersleben, Germany

**Keywords:** DArT, genetic validation, wheat, water deficit, MAS

## Abstract

Quantitative trait loci (QTL) is one of the most important steps in marker-assisted selection. Few studies have validated quantitative trait loci for marker-assisted selection of yield traits under drought stress conditions in wheat. A set of 138 highly diverse wheat genotypes were tested under normal and drought stress conditions for 2 years. Plant height, heading date, spike length, grain number per spike, grain yield per spike, and 1000-kernel weight were scored. High genetic variation was found among genotypes in all traits scored under both conditions in the 2 years. The same panel was genotyped using a diversity-array technology (DArT) marker, and a genome-wide association study was performed to find alleles associated with yield traits under all conditions. A set of 191 significant DArT markers were identified in this study. The results of the genome-wide association study revealed eight common markers in wheat that were significantly associated with the same traits under both conditions in the 2 years. Out of the eight markers, seven were located on the D genome except one marker. Four validated markers were located on the 3D chromosome and found in complete linkage disequilibrium. Moreover, these four markers were significantly associated with the heading date under both conditions and the grain yield per spike under drought stress condition in the 2 years. This high-linkage disequilibrium genomic region was located within the *TraesCS3D02G002400* gene model. Furthermore, of the eight validated markers, seven were previously reported to be associated with yield traits under normal and drought conditions. The results of this study provided very promising DArT markers that can be used for marker-assisted selection to genetically improve yield traits under normal and drought conditions.

## 1 Introduction

Wheat (*Triticum aestivum* L.) is a crop of historical importance, as it marks the turning point of human civilization 10,000 years ago with its domestication (Salamini et al., 2002). Bread wheat, an allohexaploid species, originated through two successive hybridization rounds. A second round of hybridization between tetraploid and diploid species is thought to have occurred around 10,000 years ago in the Fertile Crescent (Zhao et al., 2020; Saini et al., 2021). Since its domestication, wheat has undergone rounds of selection, adaptation, and hybridization. Today, with more than 218 M ha and almost 760 metric megatons of production, wheat is one of the most cultivated and consumed crops, providing 20% of the calorie intake per day (Sandhu K. et al., 2021). In terms of food security, wheat ranks as the second most important food crop in developing countries after rice, as around 80 million farmers rely on its production for their livelihood (Challinor et al., 2014). Wheat breeding programs mainly focus on improving grain yield, biotic and abiotic stress, and end-use quality traits (Sandhu et al., 2021c; 2022). High-yielding varieties with broader adaptations are one of the primary goals of wheat breeders globally; however, this is hindered by limited knowledge and testing of the genetic material for different agronomic and abiotic stress traits (Sallam et al., 2018; Langridge and Reynolds, 2021; Ahmed et al., 2022).

Drought affects the different physiological and yield contributing factors in the plant, which ultimately affects the plant yield (Sallam et al., 2019). Furthermore, the stage, intensity, and duration of drought conditions play an important role in deciphering its effect on the plant performance. Researchers define drought differently; most important, it is categorized as a meteorological drought, which is based on the temperature and precipitation to give an estimate of the potential evapotranspiration (PET) (Ahmed et al., 2019; Sallam et al., 2019). In wheat, depending on the environment, drought occurs when the PET is higher than the usual value for the region. The severity of the drought depends on the extent and duration of the water deficit, the vulnerability of the production system, and the limit of temperature elevation. In wheat, drought at different stages affects performances differently in different wheat-producing regions; in general, wheat is highly susceptible to drought at the flowering stage (Ahmed et al., 2019; Sallam et al., 2019). Drought occurring earlier could lead to poor establishment, which can cause tremendous or complete crop loss; on the contrary, terminal drought stress causes a reduction in the harvest index. The higher temperature throughout the growing season, which is getting common in many regions, will shorten the growth cycle, ultimately affecting the seed number and weight. Moreover, a severe heat event at critical stages also significantly affects the grain number, size, and quality (Liu et al., 2019; Tanin et al., 2022).

Connecting phenotypes with genotypes, known as genetic mapping, provides a vital tool for crop breeding and improvement (Kaur et al., 2021). Several statistical models have been developed for marker-trait associations in genome-wide association studies (GWASs), which range from simple to increasingly complex models (Saini et al., 2021). With an increase in genotyping information, statistical models that can separate the real biological association from false positives are required without the real association (false negatives) (Wang et al., 2014). False positives in models are also observed when familial relatedness or common ancestry between genotypes is not accounted for. The structure, discriminant analysis, and principal component analysis (PCA) are routinely used as a covariate in statistical models for accounting for the population structure (Price et al., 2006). However, PCA is getting more attention because of its consistent performance with structures, and it is computationally cheap to generate covariates. Identity by descent is one of the traditionally used approaches for observing familial relatedness. Recently, the kinship matrix calculated from genotyping information is used as a covariate in mixed linear models (Zhang et al., 2010).

Yield and drought tolerance are complex quantitative traits that are controlled by a large number of small- and large-effect quantitative trait loci (QTLs), and identifying all these genic regions is important for breeding for drought stress tolerance, shown in the study by Anuarbek et al. (2020) and Tanin et al. (2022). Many mapping studies have identified various QTLs for different agronomic, yield, diseases, and end-use quality-related traits in wheat using GWAS-based approaches under normal and stress conditions (Sallam et al., 2019; Sandhu et al., 2021b). Studies have been conducted to dissect the genetic architecture of yield-related traits in wheat under controlled and drought stressed conditions. MacCaferri et al. (2011) evaluated 189 elite wheat lines for 15 environments under normal and drought stress, and the number of associations under drought conditions was far less than that in normal conditions. Similarly, Sukumaran et al. (2018a) identified a large number of QTLs for stress tolerance indices, i.e., stress tolerance, stress susceptibility index, and stress tolerance index under drought and heat stress conditions. Detected and validated QTLs can be used in pre-breeding the germplasm and breeding for abiotic stress tolerant cultivars for climate resilience in wheat (Hanson et al., 2018).

In this study, we used 138 spring wheat genotypes that were phenotyped for various yield-related traits under controlled and drought conditions. The whole population was genotyped using diversity-array technology (DArT) markers using the protocol defined in the study by Saini et al. (2022). DArT markers have been used in various genome-wide association studies to identify QTLs in wheat for various traits (Saini et al., 2022). We studied the variation present among all genotypes for normal and drought stress conditions for 2 years. Association mapping was performed using the general linear model (GLM) + principal component analysis (PCA) models to account for false positives and negatives to avoid spurious associations. The QTLs identified in this study and their population were compared with other studies and breeding programs to validate the utilization of those QTLs for MAB in wheat.

## 2 Materials and methods

### 2.1 Plant material

A set of 138 highly diverse spring wheat genotypes from 22 countries was obtained from the United States Department of Agriculture (Agricultural Research Service, the GRIN-Global project), USDA. The list of genotypes and their pedigree is presented in Supplementary Table S1. The same collection was evaluated under drought stress at the seedling stage by Ahmed et al. (2021).

### 2.2 Experimental layout

In two consecutive seasons 2018/2019 and 2019/2020, all genotypes were sown under normal (N) and drought (D) conditions at the Experimental Field Station of the Department of Genetics, Assiut, Egypt, where the soil was clay loam. A randomized complete block design (RCBD) was used with two replications. From each genotype, 15 seeds were hand-sown in 1.5 m rows with 10 cm as a distance between seeds and 50 cm between rows.

For normal conditions, all genotypes were irrigated 6–7 times during growing seasons, while under drought stress, the genotypes were irrigated two times and irrigation stopped when the plant reached the tillering stage. No irrigation was applied for drought-stressed genotypes until harvesting. The soil moisture content was measured under normal and drought conditions from six soil samples taken randomly at the depth of 45 cm two times (before and after anthesis). The samples were weighed and then dried for 48 h at 110°C in an oven. The samples were then removed from the oven and weighed again, with the weight loss representing the quantity of water in the soil.

$$Soil\;moisture\;content\left(\%\right)=\left[\left(\left(Wet\;matter-dry\;matter\right)/Wet\;matter\right)\times 100\right]$$

Humidity rate = soil moisture content (percentage) × 1.2.

The heading date (HD; days) was scored as the number of days from sowing to the date when 50% of plants have started heading, and plant height (PH; cm) was scored from the ground to the tip of the main spike at maturity. Spike traits were measured including the main spike length (SL; cm), number of grains per spike (GNPS) and grain yield per spike (GYPS; g), and 1000-kernel weight (TKW; g).

### 2.3 Statistical analyses of phenotypic data

The analysis of variance was calculated for both the conditions (normal and drought) using PLABSTAT software [1], with the following model:

$$Y_{ijk}=\mu +y_{i}+r_{j}+g_{k}+gy_{ik}+yrg_{\left(ijk\right)}\left(error\right),$$

where Y_ij_ is an observation of the genotype k in year i and replication j and μ is the general mean. y_i_, r_j_, and g_k_ are the main effects of the year, replication, and genotype, respectively. The error is year × replication × genotypes interaction of genotype k with year i. Replications and years were considered as random effects.

### 2.4 Genotypic data and genome-wide association studies

A total of 407 DArT markers for the 138 genotypes evaluated in this study were downloaded from the United States National Plant Germplasm System database (https://www.ars-grin.gov/). Marker data and genotypes were filtered using the following criteria: minor allele frequency of 5% and 20% missing data. As a result of marker filtration, the remaining 398 DArT markers and 138 genotypes were used for genetic analyses.

The analysis of the population structure was performed for the same set of markers and genotypes using STRUCTURE 3.4.0 software (Pritchard et al., 2000) by Ahmed et al. (2021), who revealed that there were two possible subpopulations. Therefore, the genome-wide association analysis was performed using the general linear model with principle component analysis to correct the effect of the population structure. The GWAS was performed using TASSEL v.5.2.5 software (Bradbury et al., 2007). Marker-trait association to identify significant markers was tested at a significant level of 0.001 (Bradbury et al., 2007). The phenotypic variation explained by a marker (*R* ^2^) and the effect of the visible allele was also determined using TASSEL v.5.2.5. Linkage disequilibrium (*r* ^2^) was analyzed for the validated markers using TASSEL 5.0 v, and the haplotype (D′) view was analyzed using Haploview software (Barrett et al., 2005).

Candidate genes and their functional annotations for the validated markers were identified using Ensembl genome version 1.1 (http://ensemblgenomes.org/) using the International Wheat Genome Sequencing Consortium (IWGSC) reference sequence v1.0 to identify candidate genes and their functional annotations. Gene network for the candidate gene model detected by GWAS was analyzed from KnetMiner database (https://knetminer.com/Triticum_aestivum/).

## 3 Results

### 3.1 Genetic variations in yield traits under normal and drought conditions

**FIGURE 1:**
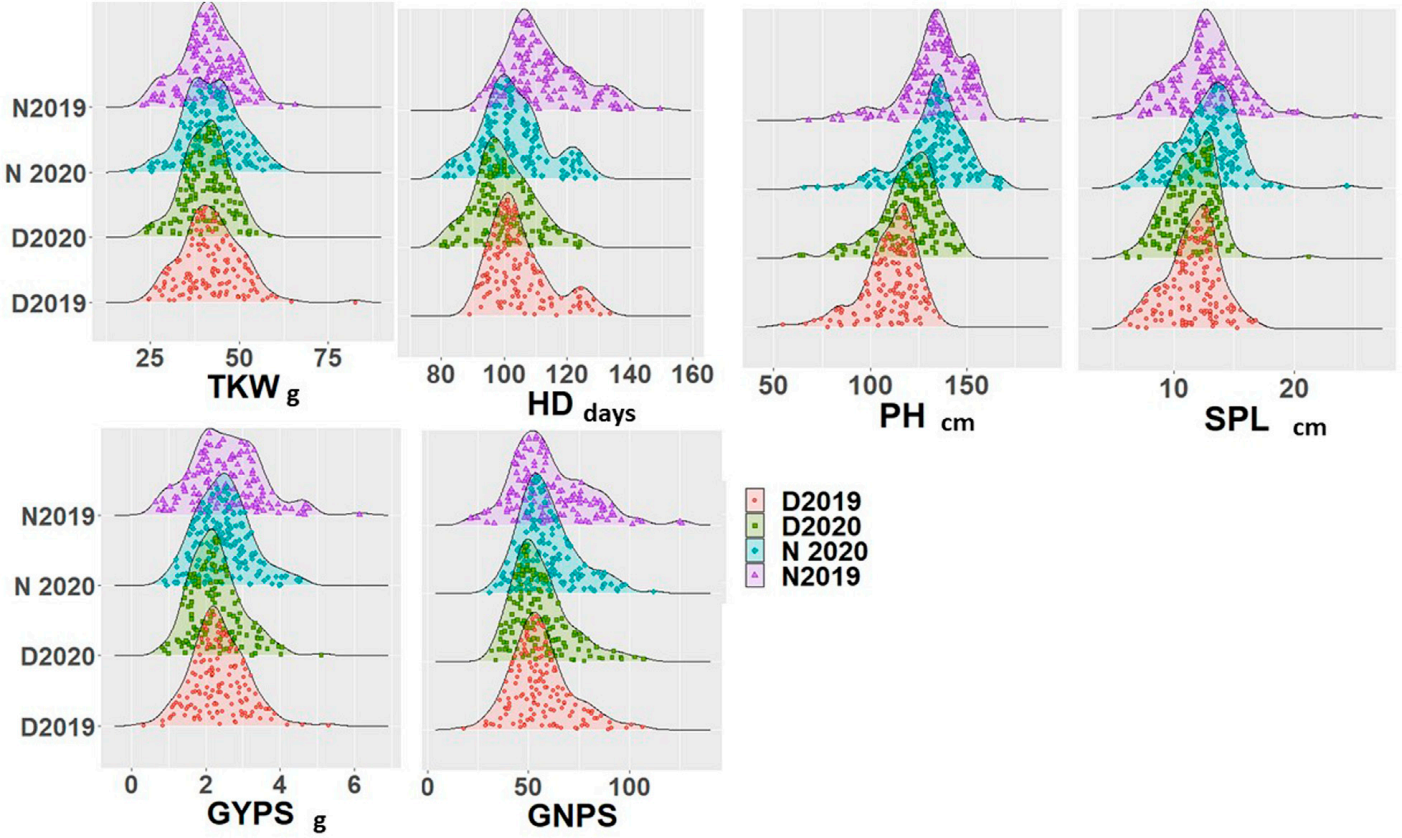
Density diagram for all genotypes under normal and drought conditions in the two growing seasons.

The analysis of variance results for all genotypes under normal and controlled conditions for both years are presented in Table 1. All the six scored traits showed significant differences at *p* < 0.05 under normal and drought stress conditions. The genotype × year interaction also showed significant differences for all traits under both conditions, except for PH under drought stress. Replication and year effects were significant for few traits under both conditions but not for all traits and stress conditions (Table 1). The phenotypic variation for all the six traits under normal and drought conditions for both years is presented in Figure 1. All the traits showed a normal distribution for both years and conditions and the results were validated with the help of the Shapiro–Wilk normality test (results not shown). Few outliers were observed for each trait under both conditions, and they are depicted in Figure 1. The average soil humidity at 10 and 35 cm depth in both conditions is presented in Supplementary Table S2.

**TABLE 1:** Analysis of variance of all traits scored under normal and drought conditions throughout the two growing seasons, 2019 and 2020.

Treat	SOV	HD	PH	SPL	GNPS	GYPS	TKW
Control	Years (Y)	555.50^a^	1.41	0.17	1.06	2.22	2.11
	Replication (R)	17.38^a^	0.56	20.32^a^	2.72	1.60	3.02
	Genotype (G)	102.69^a^	31.25^a^	23.9^a^	16.67^a^	15.26^a^	21.92^a^
	G × Y	4.44^a^	6.50^a^	7.02^a^	2.60^a^	2.03^a^	2.32^a^
Drought							
	Years	175.81^a^	101.99^a^	0.064	0.00	2.12	11.68^a^
	Replication	31.92^a^	3.31	0.01	13.50	21.20^a^	10.48^a^
	Genotype	55.32^a^	7.62^a^	11.10^a^	17.27^a^	17.28^a^	16.28^a^
	G × Y	3.16^a^	1.23	2.04^a^	3.73^a^	4.29^a^	2.21^a^

^a^ Refers to the significant level at *p* < 0.01.

### 3.2 Genome-wide association study for yield traits under normal and drought conditions

A total of 398 DArT makers obtained after filtration were used for genome-wide association studies. The number of DArT markers was 144 (36%), 162 (41%), and 58 (15%) for A, B, and D genomes, respectively. Additionally, there were 34 (8%) markers with unknown chromosomal positions. In our previous study, we showed that the whole population can be divided into two main sub-clusters using PCA and structure analysis, where clusters 1 and 2 consisted of 78 and 60 genotypes, respectively (Ahmed et al., 2021). Finally, six traits with 398 DArT markers were used for the GWAS under normal and drought stress conditions for both years. Out of the 398 markers used, about 191 significant markers were detected at the *p-*value ≤0.001 (Table 2, Supplementary Table S2). In each year, the number of significant markers detected under drought stress was larger than those detected under normal conditions. Identified markers explain 7.4%–18.7% of the total phenotypic variation for all the six studied traits (Supplementary Table S3). A set of 14 major QTLs were identified in 2019 and 60 in 2020, demonstrating a greater number of QTLs in the year 2020. The allele effect for all markers and major QTLs for all the traits is presented in Supplementary Table S3 and Table 2, respectively.

**TABLE 2:** Summary of the GWAS for yield traits under normal and drought conditions.

Traits		No. of sign. markers	*p*-value	*R* ^2^ ^a^	Allele effect	No. of major QTLs
***Season 2018/2019***						
HD	N	5	0.0003–0.0009	7.86%–8.5%	−18.9:−20.23	
	D	9	0.0001–0.00001	8.5%–12.6%	−9.7:6.78	3
PH	N	0				
	D	6	0.0001–0.0008	7.5%–9.7%	−11.8–12.9	-
SPL	N	3	0.0001–0.0008	7.8%–9.4%	1.55	-
	D	1	0.0002–0.000078	7.4%	−2.49:2.22	-
GNPS	N	2	0.0001	7.6%	15.488	-
	D	0	-	-	-	-
TKW	N	6	0.0001–0.00002	8.1%–11.8%	−6.8:9.4	4
	D	6	0.0001–0.0009	8.8%–13.0%	−5.7:8.2	4
***Season 2019/2020***						
HD	N	14	0.0001–0.00002	8.4%–15.2%	−6.3:8.3	9
	D	12	0.0001–0.00001	8.3%–13.0%	−6.9:5.9	7
PH	N	8	0.0001–0.00006	8.2%–14.6%	−12.5:17.2%	4
	D	18	0.0001–0.0000002	7.8%–18.7%	−12.6:15.1	4
SPL	N	8	0.0002–0.00006	7.8%–13.7%	−2.49:1.7	3
	D	3	0.0001–0.0008	8.0%–10.4%	−1.6:1.63	2
GNPS	N	15	0.0001–0.00000009	7.7%–19.9%	−16.13:12.07	6
	D	24	0.0001–0.0000007	7.9%–17.7%	−15.1:14.5	10
GYPS	N	18	0.0001–0.000008	7.8–13.7	−0.68:0.50	6
	D	25	0.0001–0.00001	8.2%–12.9%	−0.50:0.43	8
TKW	N	5	0.0001–0.0009	8.5%–10.3%	−4.4:6.1	1
	D	3	0.0006–0.00005	7.9%–8.8%	−3.6:4.3	-

^a^ Phenotypic variation explained by markers.

**FIGURE 2:**
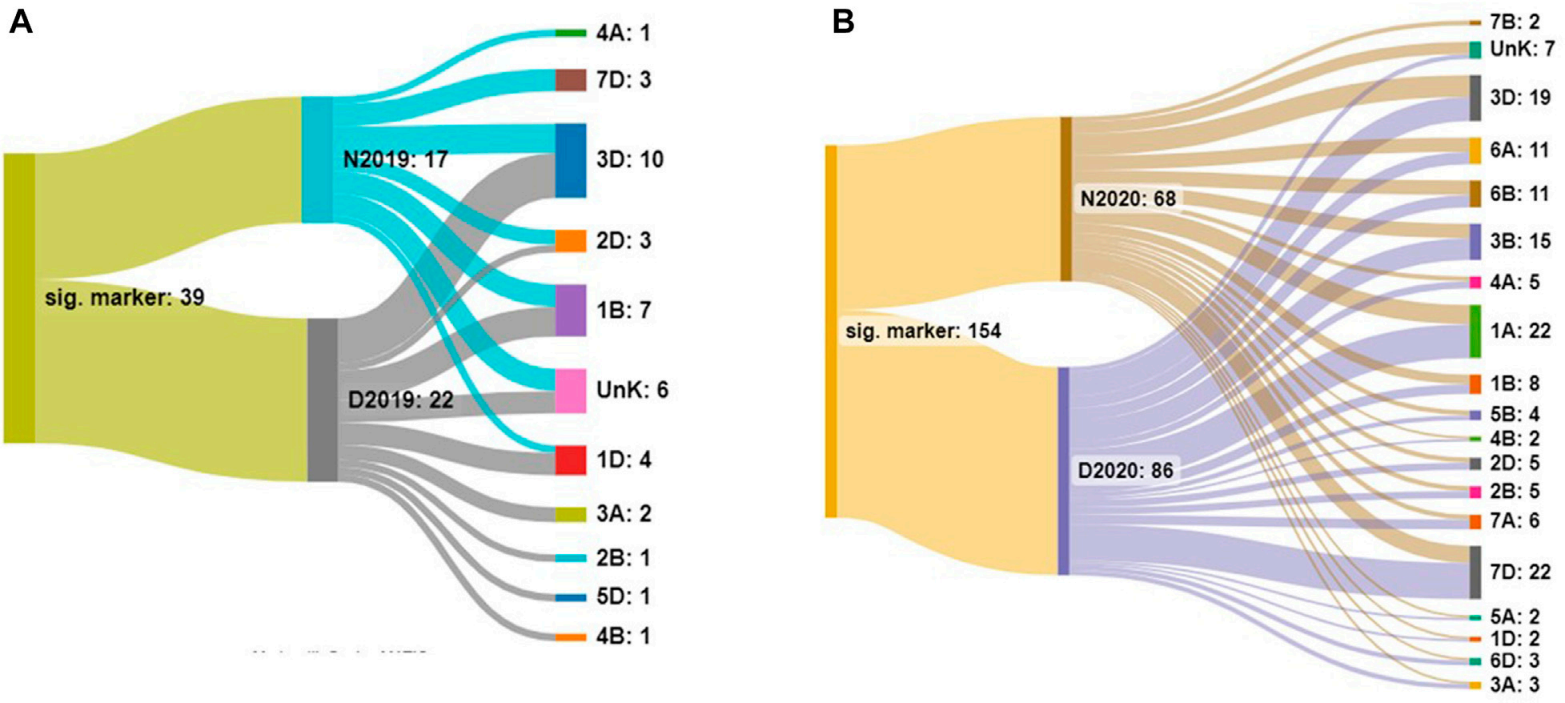
Number of significant markers in each environment and on each chromosome under normal (N) and drought (D) conditions in 2019 **(A)** and 2020 **(B)**.

The number and distribution of identified markers on different chromosomes for both years under normal and drought stress conditions are presented in Figure 2. Significant markers were located on 11 chromosomes, 1A, 1D, 2B, 2D, 3A, 3D, 4A, 4D, 7D, and unknown, in the 2 years. The highest number of significant DArT markers were found on 3D and 7D chromosomes in 2019/2020 and 2020/2021, respectively.

For HD, three, nine, and seven major QTLs were identified under normal conditions in 2019, normal conditions in 2020, and drought stress conditions in 2022, respectively. *R* ^2^ for these QTLs varied from 7.86%–15.2% for the phenotypic variation for HD (Supplementary Table S2 and Figure 2). No QTL was identified for PH in 2019, while four QTLs were identified each for normal and drought stress in 2020. These eight QTLs explained 7.8%–18.7% of the phenotypic variation for PH (Table 2; Figure 2). For SPL, three, three, and two QTLs were identified under drought conditions in 2019, normal conditions in 2020, and drought stress conditions in 2022, respectively. *R* ^2^ for these QTLs varied from 7.8%–13.7% for the phenotypic variation for SPL. The GNPS was associated with six and ten QTLs under normal and drought conditions for 2020. These QTLs explained 7.7%–19.9% of the phenotypic variation for the GNPS. The TKW was associated with nine markers, from which four were present under normal conditions in 2019, four under drought stress in 2019, and the last one under normal conditions in 2020. *R* ^2^ for these QTLs varied from 8.1%–13.0% for the phenotypic variation for the TKW.

**FIGURE 3:**
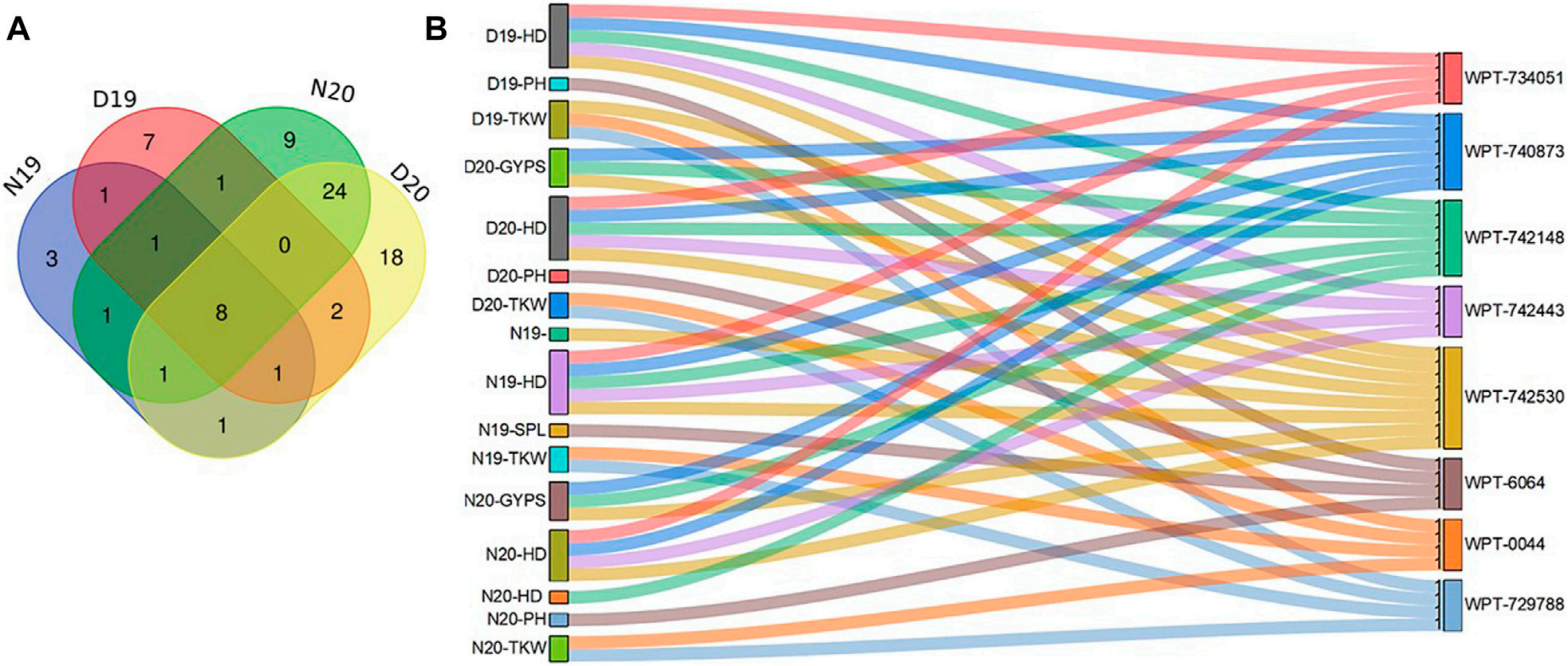
Venn diagram for significant markers in both conditions in the 2 years **(A)** and validated markers with pleotropic effects **(B)**.

Interestingly, markers having associations with more than one trait are presented in Supplementary Table S4. There were 11 common markers that were identified for normal and drought stress conditions for the year 2019, while 33 were common for the year 2020 (Figure 3A). Furthermore, there were 11 common markers identified for both years under normal conditions, and 11 were common in both years for drought conditions. Some markers had significant associations with the same trait under normal conditions in both years (N19 and N20), such as WPT-9196, which was associated with the GNPS. Likewise, in both years, some markers, such as WPT-742230, were found to be significantly associated with the TKW only under drought conditions (D19 and D20).

Notably, a total of 44 markers were found to be associated with more than on trait, ranging from two to eight traits (Supplementary Table S4). The WPT-1786 marker was found to be associated with eight traits under N19, N20, D19, and D20. Three markers WPT-6064, WPT-0044, and WPT-729788 were found to be associated with PH and TKW in D19 and D20, respectively. Some markers were found to be associated with the same traits under all conditions.

Finally, there were eight common markers that were present under both years and both conditions (normal and drought) (Figure 3A). In our study, we focused on the common markers that had significant associations under normal and drought stress in both years.

### 3.3 Validation of the DArT-genomic region associated with yield traits

**FIGURE 4:**
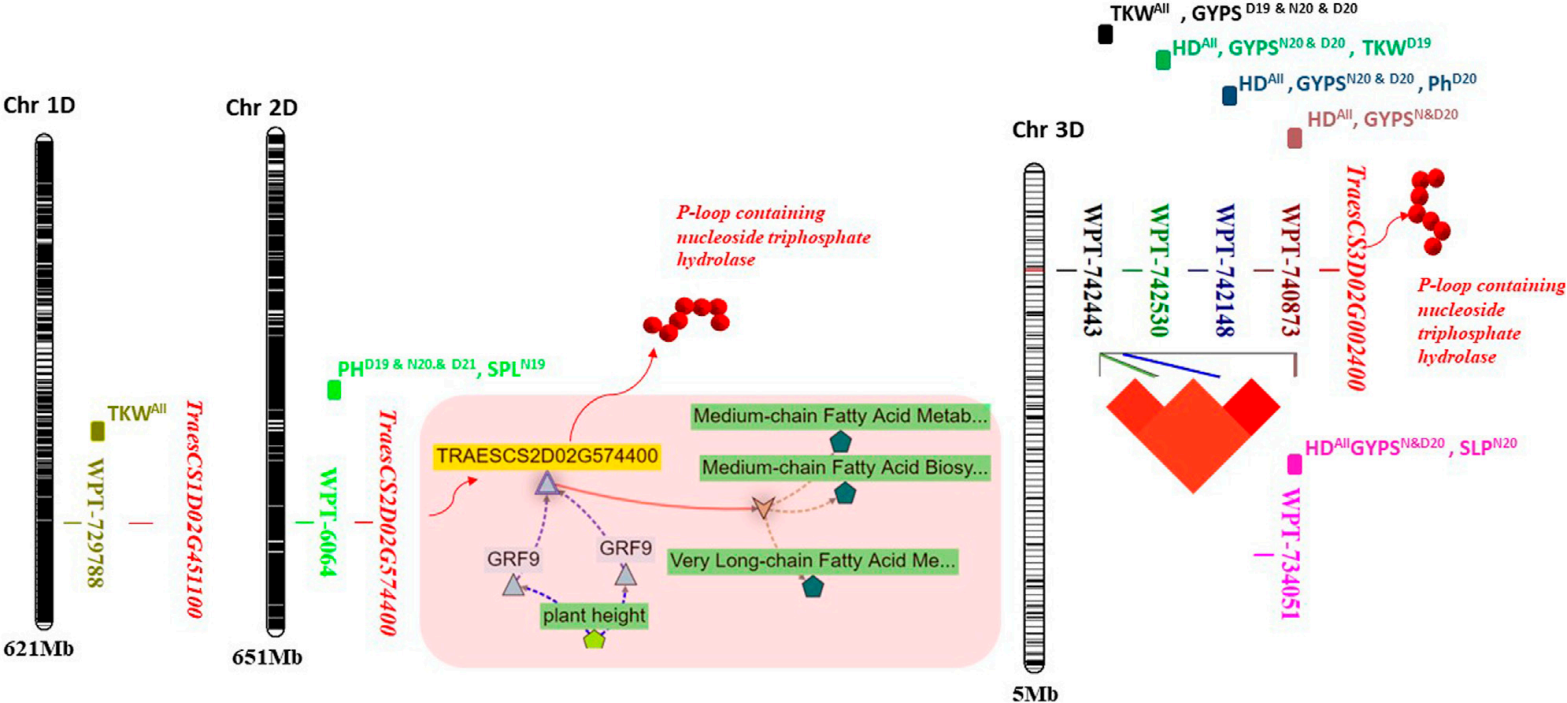
Physical position of the validated significant markers located on 1D, 2D, and 3D chromosomes. The gene network for the TraesCS2D02G574400 gene model was illustrated on the 2D chromosome. The linkage disequilibrium among the four markers on 3D was illustrated on the 3D chromosome.

The eight markers that had significant associations with yield traits are presented in Table 3 and Figure 4. Interestingly, most of these markers were located on the D genome (Figure 4). The haplotype analysis for these markers is presented in Supplementary Figures S1–S3. The WPT-729788 marker was located within the TraesCS1D02G451100 gene model, which encodes to protein EARLY FLOWERING 3-like. WPT-6064 was found to be associated with PH in D19, D21, N20, and with SPL in N19. This marker was located with TraesCS2D02G574400, which encoded a P-loop containing nucleoside triphosphate hydrolase (Figure 4). Notably, four markers were found to be associated with HD under both conditions in the 2 years. These four markers were found in complete linkage disequilibrium and were located within a TraesCS3D02G002400 gene model which encodes a P-loop containing nucleoside triphosphate hydrolase. The present allele of all the four markers was found to be associated with early flowering. Among the four markers, WPT-734051 had a major effect on HD (*R* ^2^ <10%) under D19, N20, and D20 (Supplementary Table S5). Three DArT markers WPT-0044, WPT-729788, and WPT-742443 were found to be significantly associated with the TKW under both conditions in the 2 years. Present alleles of the three markers were associated with a decreased TKW. Two markers WPT-742443 and WPT-6064 were found to be associated with the GYPS and PH under drought conditions in the 2 years, respectively. No candidate gene models were found for WPT-0044.

**TABLE 3:** List of validated DArT markers associated with yield traits under normal (N19 and N20) and drought (D19 and D20) conditions.

Validated markers	Chro: Pb	N19	D19	N20	D20	References	Candidate gene	Protein coding
WPT-729788	1D: 493482175–493482606	TKW	TKW	TKW	TKW	Spike number per plant (Cui et al., 2013)	-	-
WPT-6064	2D: 639091664–639092067	SPL	PH	PH	PH	Plant height (http://knetminer.org/data/rdf/resources/trait_to_0000207)	TraesCS2D02G574400 (*RGA5*)	P-loop-containing nucleoside triphosphate hydrolase
WPT-742443	3D: 1122666–1123082	HD	HD	HD and GYPS	HD and GYPS	Grain yield (Atta 2013)	TraesCS3D02G002400	P-loop-containing nucleoside triphosphate hydrolase
WPT-742530	3D: 1122666–1123082	HD	HD and TKW	HD and GYPS	HD and GYPS	Grain yield and grain protein content (Atta 2013)	TraesCS3D02G002400	P-loop-containing nucleoside triphosphate hydrolase
WPT-742148	3D: 1122745–1123008	HD	HD	HD and GYPS	HD, PH, and GYPS	Spike number per plant (Cui et al., 2013)	TraesCS3D02G002400	P-loop-containing nucleoside triphosphate hydrolase
WPT-740873	3D: 1122645–1123102	HD	HD	HD and GYPS	HD and GYPS	Spike number per plant (Cui et al., 2013)	TraesCS3D02G002400	P-loop-containing nucleoside triphosphate hydrolase
WPT-734051	3D: 4166079–4166677	HD	HD	HD	HD	Spike weight (Ogbonnaya et al., 2017)	-	-
WPT-0044	1B	TKW	TKW	TKW	TKW	-		

Out of the eight markers, five these markers were found to be associated with more than one trait. For example, the high-LD genomic region found on the 3D chromosome had associations with HD (all conditions), GYPS (N20 and D20), TKW (D19), and PH (D20). Furthermore, out of the eight significant markers, seven were reported in earlier studies with their significant associations with yield traits under normal and drought conditions (Table 3).

## 4 Discussion

Important abiotic stresses affecting the wheat yield includes heat, drought, salinity, mineral toxicity, and waterlogging (Erdayani et al., 2020; Mourad et al., 2021; Amro et al., 2022). Drought affects 42% of the wheat production area, while heat affects 58% (Tanin et al., 2022). Climatic uncertainty causes warmer temperatures, and erratic rainfalls are predicted in the future, which could potentially convert the mega-productive environment to short-season drought stress environments (Mourad et al., 2019; Mondal et al., 2021). These conditions represent a unique challenge to plant scientists for releasing climatic resilient cultivars (Sallam et al., 2019). Furthermore, drought is a polygenic trait controlled by a large number of genes and, hence, is difficult to understand (Sukumaran et al., 2018b).

We phenotyped six different yield-related traits, i.e., HD, PH, SPL, GNPS, GYPS, and THW, under normal and drought stress. Significant differences were found between soil water capacities in both conditions in the 2 years, indicating that the genotypes under drought conditions were exposed to a water deficit. A high genetic variation existed among genotypes in all traits that can be exploited for phenotypic selection under drought stress. The genotype × year interaction was also significant for these traits, which can be attributed to the interaction of genotypes with management-, environmental-, and year-related conditions, thus suggesting the need to evaluate the lines under multiple environments and years for studying drought-related traits. These traits showed a normal distribution under both the conditions and years, which provides an opportunity to make a selection from both sides of the distribution according to the need of the breeding program and expected stress conditions, which is pretty common in quantitative traits as reported in the literature (Bhatta et al., 2018; Sallam et al., 2019). Moreover, under the drought conditions, the means of all these six traits were less than normal conditions and similar trends were reported for durum, spring, and winter wheat varieties (Liu et al., 2019). The soil moisture volume was 19% at a 10 cm depth and 35% at a 35 cm depth. In wheat, the optimum volumetric soil-moisture content remaining at field capacity is about 45%–55% [three feet below the soil surface for clay soils and it is 15%–20% in the wilting stage (https://nrcca.cals.cornell.edu/soil/CA2/CA0212.1-3.php)], which is defined as the soil water content when plants growing in that soil wilt and fail to recover their turgor upon rewetting, indicating that successful drought stress occurred in the population in the 2 years.

### 4.1 Genome-wide association study

The GWAS was performed for all traits using DArT markers that were widely and previously used to identify important QTLs for target traits such as disease resistance in wheat (Ladejobi et al., 2016; Mourad et al., 2021). Moreover, many earlier studies have used DArT markers for identifying genomic regions associated with drought tolerance and yield trait studies in wheat (Ovenden et al., 2017; Ahmed et al., 2021). These DArT markers have become available in the United States National Plant Germplasm System database for many wheat genotypes. Therefore, utilizing such an important genotypic database for genetic association analyses will be fruitful for marker-assisted selection to improve target traits through the validation of previously reported QTLs in different genetic backgrounds (Alexander et al., 2012; Choudhury et al., 2021; Sallam et al., 2023).

The high genetic variation found among genotypes was very useful to identifying important significant DArT markers. The same population with the same number of DArT marker was used to identify important genomic regions associated with drought tolerance at the seedling stage (Ahmed et al., 2021). For GWAS studies, 100–500 identical markers should be used to detect the potential marker-trait association (Yu et al., 2012; Alqudah et al., 2020). Although the number of DArT markers used in this study was 407, they were distributed in all wheat chromosomes. As mentioned previously, the same DArT markers were previously used for yield traits under drought and normal conditions. Therefore, the main target of our study was to test the association of some previously reported markers in our current wheat panel. Moreover, the same population and same marker number were used to identify genomic regions associated with stripe rust in wheat (Mourad et al., 2021).

Accounting for the population structure within the mapping population is a critical step before performing GWA and mapping studies (Price et al., 2006; Zhang et al., 2010). It provides an idea of the genetic relationship among the lines present in the population and assists in identifying genetic diversity in the target population to control for spurious associations. False positives in GWA models are also observed when familial relatedness or a common ancestry among the genotypes is not accounted for (Liu et al., 2016). Structure, discriminant analysis, and principal component analysis are routinely used as a covariate in statistical models for accounting for the population structure. Our previous study showed that this population is subdivided into two clusters based on the results from STRUCTURE software and PCA (Ahmed et al., 2021). Within the cluster, genotypes were categorized based on their country of origin and responses to drought conditions. The same testing population was genotyped using SNP markers in another study by Mourad et al. (2020), and PCA showed the same subculturing techniques obtained using DArT markers in this study and, thus, showed the effectiveness of using DArT markers. These results gave us confidence in understanding the structure present in our testing population and we accounted for that using PCA in the GLM for conducting genome-wide analyses.

The GWAS is one of the most used methods for complex quantitative traits, especially drought, in this study (Kaur et al., 2021). Most of the traits controlling drought are complex and controlled by a large number of small-effect genes (Mathew et al., 2019).

In this study, out of the 398 markers used, about 191 significant markers were detected at a *p*-value ≤0.001, which was significantly associated with various target traits. We were able to identify 14 major QTLs for 2019 and 60 major QTLs for 2020 datasets for normal and drought conditions. The majority of these QTLs and markers were identified under drought stress conditions, thus showing that these markers have a strong association with traits scored under drought stress and can ultimately be used for breeding drought tolerance in wheat (Mourad et al., 2018; Ahmed et al., 2021). Different numbers of QTLs were identified for each trait for each year and stress condition, which can be correlated to the response of these genotypes differently to drought stress conditions. There were 11 common markers identified for both years under normal conditions, and 11 markers were common in both years for drought conditions, which suggest the heritability of these markers and their expressions under different environmental and year conditions. Moreover, a set of 71 markers were found to have major effects with *R* ^2^> 10% on yield traits under both conditions. Also, many markers were found to have a significant association with more than one trait, indicating that these markers showed pleotropic effects. For example, the WPT-1786 marker was found to be associated with PH, GNPS, HD, and GYPS under N20 and D20. Under drought conditions in both years, three markers were found to be associated with HD. One marker, WPT-6064, was found to be associated with PH. Two markers were found to be associated with the TKW. Such markers could be very useful genomic regions, as they remained significant with the same traits in both the years under drought stress. Also, they could be considered as validation markers for the respective trait under certain conditions. Genetic validation of a QTL can be carried out when the same QTL or gene tends to be significantly detected when the material is grown in other years (Sallam et al., 2023).

### 4.2 Genetic validation of QTLs controlling yield traits under drought stress

QTL/marker validation is an important step for molecular breeding to improve target traits and marker-assisted selection. A set of eight common markers were present under both years and both conditions (normal and drought). Seven of these markers were located on the D genome, indicating that this genome may include important genome regions for drought tolerance. Five markers were located on the 3D chromosome. Of these five markers, four were found in complete LD (1122666–1123102 bp), indicating that the four markers within this genomic region seem to have been co-inherited together. The genomic region, including these four markers, was located within a TraesCS3D02G002400 gene model which encodes a P-loop containing nucleoside triphosphate hydrolase. The protein has a role in zinc-ion binding (Jadon et al., 2023). It plays a role in Zn, Fe, and protein remobilization in seeds during grain development (Distelfeld et al., 2006; Ricachenevsky et al., 2013) and in nitrogen from vegetative tissues to grains (Waters et al., 2009). So, these genes have a molecular function which is important to improving the yield grain quality. All four markers located in this gene were found to be associated with increased the GYPS and TKW. Moreover, the four makers were associated with HD under all conditions, suggesting that this genomic region is important for improving early flowering in wheat, which is an important trait for wheat breeders. Interestingly, these four markers were previously reported to be significantly associated with the grain yield, number of spikes/areas, grain protein content, and spike number per plant (Cui et al., 2014). WPT-734051 was found to be associated with HD in this study and with the spike weight in the study by Ogbonnaya et al. (2017). The WPT-6064 marker was associated with SPL (N19) and PH (D19, N20, and D20). This marker located within the TraesCS2D02G574400 (*RGA5*) gene model encodes the same protein found in TraesCS3D02G002400. This further supports the importance of this protein in improving yield traits under normal and drought conditions. The gene network of TraesCS2D02G574400 is presented in Figure 4. It seems that this gene is regulated by the *GRF9* gene, which is strongly associated with the plant height. Furthermore, this gene is present in a biological process which contributes to the medium-chain fatty acid biosynthetic process that plays an important role in the spike and seed formation (Mihálik et al., 2015). WPT-729788 was found to be significantly associated with the TKW in all conditions and with the spike number per plant in the study by Cui et al. (2013). The three important features of these eight markers found in this study are as follows: 1) they were associated with the same trait under normal and drought conditions in the 2 years, 2) they were previously reported in other studies, and 3) they have pleiotropic effects. Therefore, these markers can be useful in breeding programs for MAS for improving yield traits under normal and drought tolerance conditions in wheat. This can be considered as QTL validation, as genetic validation examines whether the same marker/QTL is significantly detectable when the plant material is tested in other locations or years and whether its effect on the same marker can still be detected when examined in different genetic backgrounds (Sallam et al., 2023). Here, we detected the same marker in all tested conditions and also examined the same marker in a different genetic background that was completely different from those that were previously reported.

## Data Availability

The original contributions presented in the study are included in the article/Supplementary Material; further inquiries can be directed to the corresponding author.
