# Supplementary material for: Validation and marker-assisted selection of DArT-genomic regions associated with wheat yield-related traits under normal and drought conditions
Source: Front Genet. 2023 May 23;14:1195566. doi: 10.3389/fgene.2023.1195566 (PMC10245129; doi:10.3389/fgene.2023.1195566)
Supplement: Supplementary file 1 [file Presentation1.PPTX]

## Slide 1
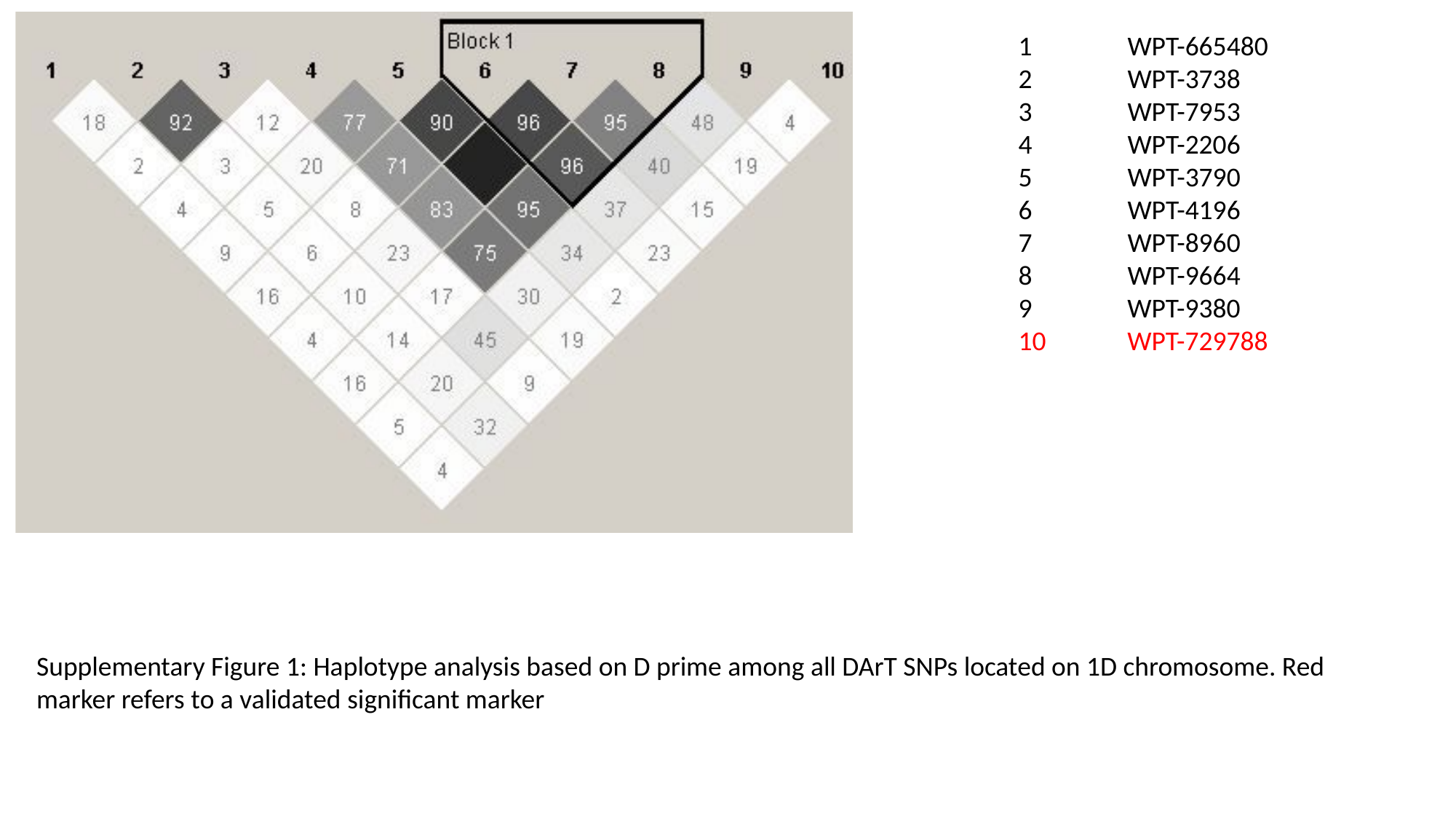

1	WPT-665480
2	WPT-3738
3	WPT-7953
4	WPT-2206
5	WPT-3790
6	WPT-4196
7	WPT-8960
8	WPT-9664
 WPT-9380
 WPT-729788
Supplementary Figure 1: Haplotype analysis based on D prime among all DArT SNPs located on 1D chromosome. Red marker refers to a validated significant marker

## Slide 2
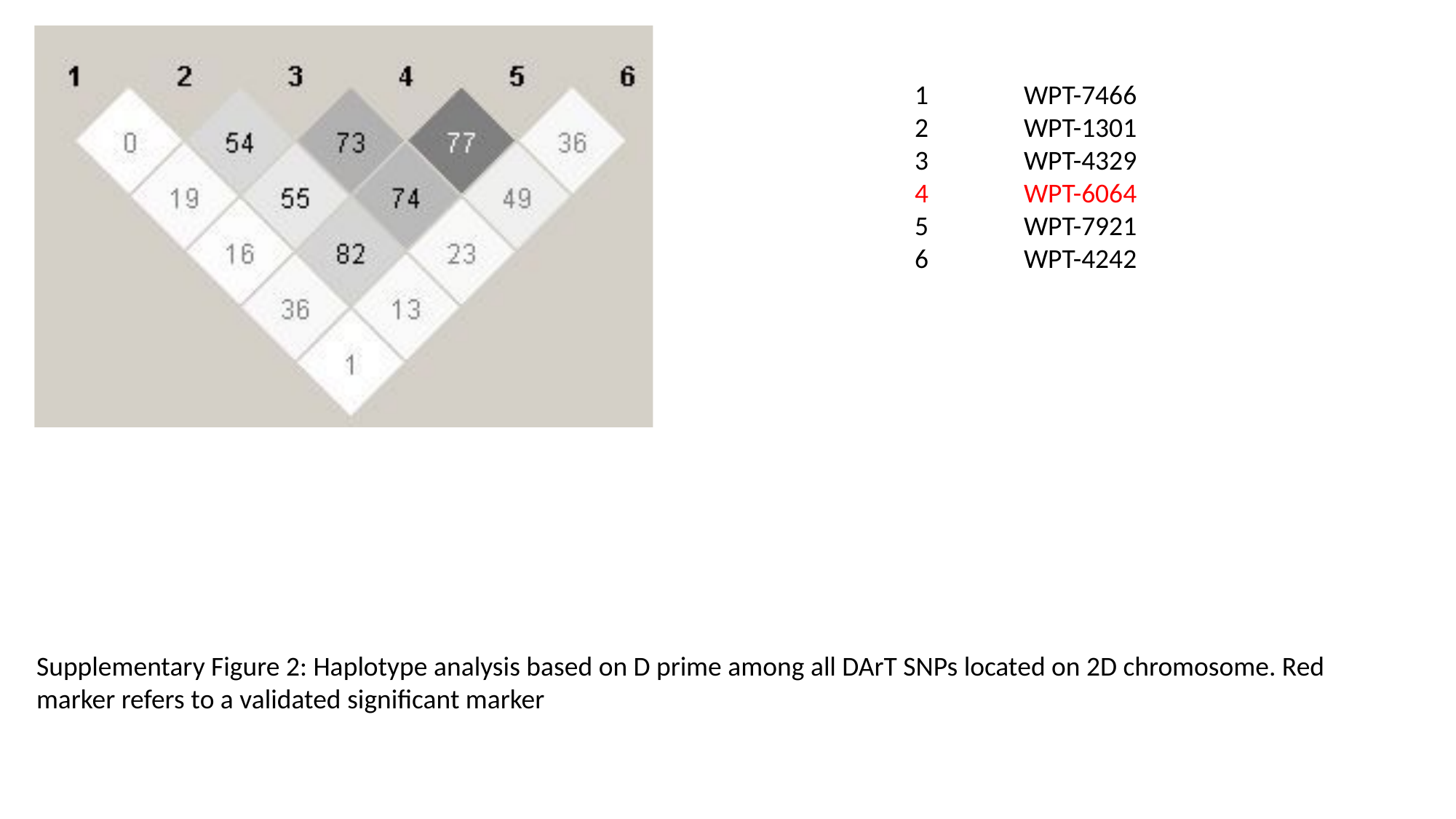

1	WPT-7466
2	WPT-1301
3	WPT-4329
4	WPT-6064
5	WPT-7921
6	WPT-4242
Supplementary Figure 2: Haplotype analysis based on D prime among all DArT SNPs located on 2D chromosome. Red marker refers to a validated significant marker

## Slide 3
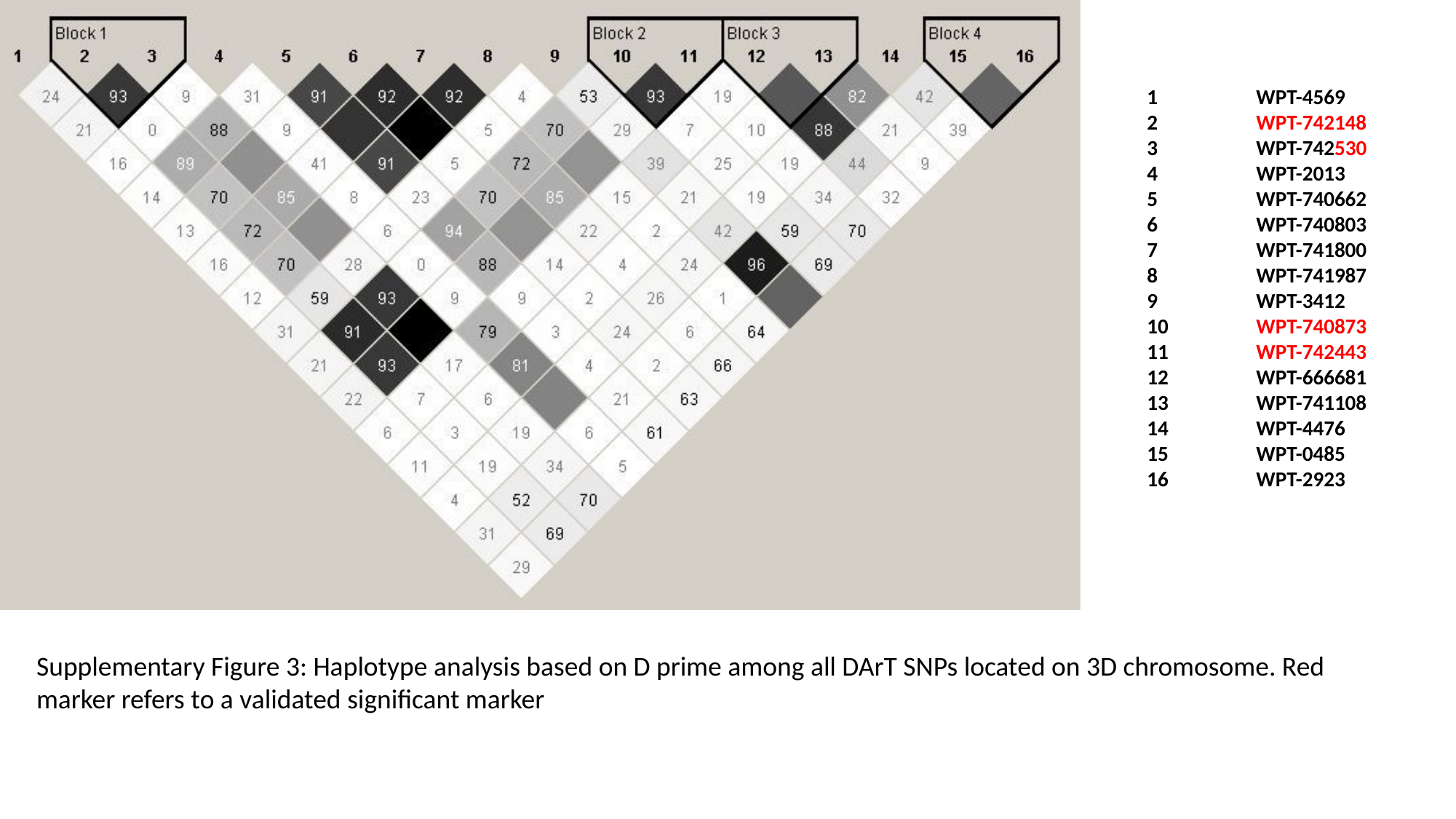

1	WPT-4569
2	WPT-742148
3	WPT-742530
4	WPT-2013
5	WPT-740662
6	WPT-740803
7	WPT-741800
8	WPT-741987
9	WPT-3412
10	WPT-740873
11	WPT-742443
12	WPT-666681
13	WPT-741108
14	WPT-4476
15	WPT-0485
16	WPT-2923
Supplementary Figure 3: Haplotype analysis based on D prime among all DArT SNPs located on 3D chromosome. Red marker refers to a validated significant marker
